# Ablative Techniques for Sarcoma Metastatic Disease: Current Role and Clinical Applications

**DOI:** 10.3390/medicina59030485

**Published:** 2023-03-01

**Authors:** Evgenia Efthymiou, Georgios Charalampopoulos, Georgios Velonakis, Stauros Grigoriadis, Alexis Kelekis, Nikolaos Kelekis, Dimitrios Filippiadis

**Affiliations:** 2nd Department of Radiology, University General Hospital “ATTIKON”, Medical School, National and Kapodistrian University of Athens, 12462 Athens, Greece

**Keywords:** sarcoma, ablation, oligometastatic

## Abstract

Sarcomas are heterogenous mesenchymal neoplasms with more than 80 different histologic subtypes. Lung followed by liver and bone are the most common sites of sarcoma metastatic disease. Ablative techniques have been recently added as an additional alternative curative or palliative therapeutic tool in sarcoma metastatic disease. When compared to surgery, ablative techniques are less invasive therapies which can be performed even in non-surgical candidates and are related to decreased recovery time as well as preservation of the treated organ’s long-term function. Literature data upon ablative techniques for sarcoma metastatic disease are quite heterogeneous and variable regarding the size and the number of the treated lesions and the different histologic subtypes of the original soft tissue or bone sarcoma. The present study focuses upon the current role of minimal invasive thermal ablative techniques for the management of metastatic sarcoma disease. The purpose of this review is to present the current minimally invasive ablative techniques in the treatment of metastatic soft tissue and bone sarcoma, including local control and survival rates.

## 1. Introduction

Mesenchymal neoplasms constitute a rare entity, characterized by highly histological and molecular heterogenicity, rendering the diagnosis, classification and treatment of these tumors challenging. The recent publication of the new WHO classification of Soft Tissue and Bone Tumors identifies more than 80 different histologic subtypes and thus, highlights the vast spectrum of mesenchymal diseases [1]. Soft tissue sarcomas (STS) have an estimated incidence of 4–5/100.000/year (excluding GIST), with liposarcomas and leiomyosarcomas being the commonest types encountered [2,3]. Bone sarcomas account for <0.2% of all malignant tumors, with osteosarcoma, chondrosarcoma and Ewing sarcoma having the highest prevalence among all the subtypes [4]. Previous radiation therapy to the affected area, exposure to various chemicals (e.g., herbicides, such as agent orange) and underlying genetic syndromes (e.g., Li-Fraumeni syndrome or neurofibromatosis) constitute major risk factors for these tumors [5]. Furthermore, approximately 20–50% of the patients with soft tissue sarcomas and 30–50% of patients with osteosarcomas will develop metastatic disease [6,7]. Lung is the primary site of metastasis followed by liver and bone [2,4]. Systemic therapy remains the standard of care for advanced sarcoma disease, whereas surgery has emerged as an effective curative approach for oligometastatic disease [2,4,7,8].

According to the current guidelines, surgery is the standard of care in patients with localized STS, aiming for R0 or if not feasible R1 resection; systemic therapy is recommended for advanced and metastatic STS [3,4,5]. First line systemic therapies are relatively standard in different sarcoma histologic subtypes including doxorubicin, combination of doxorubicin with olarutumab or dacarbazine as well as multi-agent therapies with adequate-dose anthracyclines plus ifosfamide leading to higher tumor response rates and a prolonged progression free survival [3,4,5]. Neurotrophic tyrosine receptor kinase (NTRK) inhibitors are standard treatment of patients with advanced NTRK-rearranged sarcomas; NTRK could be considered also in the preoperative setting, when a cytoreduction can improve morbidity and function [3,4,5]. Second-line and beyond therapies depend on the sarcoma histologic subtype; Gemcitabine/Docetaxel, Pazopanib and Trabectedin have been suggested for several subtypes of sarcomas [3,4,5]. New treatment options include targeted therapeutic agents such as Regorafenib, Selinexor and agents of epigenetic pathway [3,4,5].

Stereotactic body radiation therapy (SBRT) or hypofractionated radiation therapy in sarcoma metastatic disease aim to reduce tumor burden, provide local control, or symptoms’ palliation, and potentially prolong survival [9]. Apart from SBRT, brachytherapy in sarcoma disease has been used either as monotherapy in low-risk cases or as a brachytherapy boost in cases with higher risk of recurrence; however, current data regarding the effectiveness of brachytherapy in metastatic sarcoma disease remain limited [10].

Interventional radiology and specifically ablative techniques (including radiofrequency ablation, microwave ablation and cryoablation) have been recently added as an additional alternative curative or palliative therapeutic tool in sarcoma metastatic disease, concerning mainly lung, liver, and bone metastases, in combination with the systemic therapies. The publication by the French Sarcoma Group evaluating oligometastatic sarcoma patients who underwent a combined approach of both systemic and locoregional therapies (on terms of surgery or percutaneous ablation) constitutes a landmark paper providing the evidence necessary for a paradigm shift in the management of this patient population [11]. The rationale for proposing ablative over surgical approaches in sarcoma metastatic disease include the potential of decreased recovery time, preservation of the treated organ’s long-term function, minimal invasiveness, and the ability to be performed in patients deemed not medically fit for surgery [12]. Along with the vast development of thermal ablation techniques, irreversible electroporation (IRE) a non thermal method has emerged as a new promising treatment option in primarily inoperable tumors and locally advanced cancers. Nevertheless, the role of IRE in metastatic sarcoma is yet to be established as there are only a few cohort data available in the current literature. The purpose of this review is to present the current minimally invasive ablative techniques in the treatment of metastatic soft tissue and bone sarcoma, including local control and survival rates.

## 2. Thermal Ablation of Lung Metastasis

**Figure 1:**
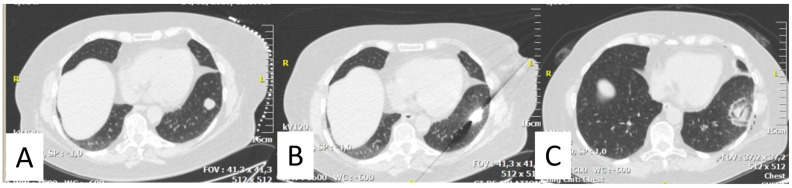
(**A**) 59 years-old female sarcoma patient with a solitary metastasis of the left lung; (**B**) CT-guided microwave ablation was performed; (**C**) Metastasis was completely ablated with safety margins (ground glass infiltrate).

Minimally invasive image-guided thermal ablative therapies have emerged as an alternative curative option for the treatment of oligometastatic pulmonary disease. In the current literature there is a growing number of studies demonstrating high local tumor control, prolonged survival rates, along with low complication rates and shorter hospital stay [13]. The ideal target for percutaneous ablation in the lung is a lesion with diameter <3 cm, fully surrounded by non-neoplastic aerated lung parenchyma. The size of the lesion (<2 cm), the number, and the location along with neoplasmatic substrate and disease-free interval constitute significant success factors [14,15]. Different ablative techniques offer distinctive advantages, and the optimal method has yet to be determined. When RFA is the ablative method of choice expandable monopolar probes are highly recommended as they are less prone to migration [13]. Microwave ablation (MWA) on the other hand is less severely affected by heat-sink effect and related to a faster and more intense heating of the tissue (Figure 1), [13]. Additionally, while both methods present comparable efficacy, MWA is better tolerated and is usually considered more suitable for larger tumors [13,15]. Cryoablation (CA) has also been proven a similarly efficacious and safe technique with the significant advantages of direct ice ball visualization during the procedure and the capability to be performed under local anesthesia [16,17]. However, cryoablation is related to higher cost and longer procedural time [13,16].

Metastasectomy is the standard of care in metachronous lung tumors in soft tissue sarcomas, with a disease-free interval of more than one year [2,7]. Similarly in pulmonary metastatic disease from bone sarcomas, the combination of surgery and systemic therapy has proven to prolong survival [4,8]. Thermal ablation techniques are recommended as an effective alternative treatment in selected cases [2,7,8]. At present, there is a limited number of studies addressing minimally invasive ablative techniques for lung metastases exclusively from soft tissue and bone sarcoma. In a large patient series by de Baere et al. evaluating 566 patients with 1037 metastases of various neoplasmatic substrates, the 1-, 3- and 5-years overall survival rates in the sarcoma subgroup (51 patients) were 94.1%, 58% and 41.5% respectively; the 1-, 3- and 5-years progression free survival and treatment failure rates were 43%, 26.5%, 15.9% and 6.1%, 8.3% and 8.3% respectively [14]. Nevertheless, the number of studies showing promising results in terms of recurrence and survival rates is increasing. A large Japanese study evaluated retrospectively the intermediate and long-term results of percutaneous computed tomography (CT)—guided radiofrequency ablation in 46 patients (144 lesions) with metastatic lung disease from bone and soft tissue sarcomas [18]. The mean lesion size was 1.3 cm and the overall survival rates were 80.6%, 70.1%, and 47.1% for 1-, 2-, and 3-years follow-up respectively; in the same series primary and secondary efficacy rates were 83.5% and 90.0% at 1 year and 76.3% and 81.4% at 2 years respectively. Palussière et al. [19] performed CT guided RFA in 29 patients (47 lesions with a mean tumor diameter of 9 mm) and demonstrated overall survival rates of 92.2% and 65.2% in 1 and 3 years respectively. Similar results were reported by Nakamura et al. [20] who achieved a local control rate of 90% and a 2-year overall survival rate of 70% for GIST and 40% for non-GIST tumors. Yevich et al. applied percutaneous CT-guided RFA in oligometastatic pediatric patients (11 children with 26 lung metastases) with pulmonary sarcoma metastases recurring after a previous surgical resection [21]. During a median follow-up of 16.7 months, 100% local tumor control was achieved. Five patients remained in complete remission during a median follow-up of 37.5 months and five patients developed new metastases including one bone and one lung metastases. Two of the five patients were retreated and were still in remission after subsequent treatment at the time of publication.

The efficacy of other thermal techniques including microwave ablation and cryoablation in pulmonary sarcoma metastatic disease has not been extensively addressed. Bourgouin et al. evaluated 27 patients (67 lesions) who underwent either CA or MWA ablation for pulmonary sarcoma metastatic disease demonstrating a high primary technical success and local tumor control, as well as overall survival rates for both ablative techniques, especially for tumors 1 cm or smaller [22]. Furthermore, there was no statistically significant difference reported concerning local progression related neither to the two ablation modalities nor to the location of the lesions (whether peripheral or non-peripheral).

Overall, thermal ablation techniques in pulmonary sarcoma metastatic disease have been reported highly efficacious, demonstrating survival rates comparable to surgery [23,24,25,26]. Additionally, percutaneous techniques offer the advantage of low non—life threatening complication rates and sparing of surrounding lung parenchyma [6]. Pneumothorax is the commonest side effect reported followed by hemoptysis. Moreover, the advantages of repeatability and combination with surgery as well as available systemic therapies renders these techniques significantly attractive to current practice [20].

Thermal ablation procedures, as acknowledged by de Baere et al. [25] like metastasectomy, aim to create a safe disease-free margin around the targeted lesions and a well-tolerated post procedural period. As in pulmonary metastasectomy negative margins, increased size, and number of the lesions as well as bilaterality, are factors significantly affecting the outcome in percutaneous ablation. In most available studies, a lesion size >20 mm and proximity to large vessels (<3 mm) have been strongly associated with local recurrence [19,27] whilst incomplete ablation has been also correlated with a negative outcome [19]. De Baere et al. highlighted the significance of wide ablation margins of 10 mm, as local tumor control rates were decreased from 93% to 80% when these margins could not be reached [25]. Moreover, similarly to surgery, a disease-free interval between the diagnosis of the primary tumor and lung metastases has been correlated with overall survival [26]. Local control of the primary sarcoma, as well as sex, age, tumor laterality, tumor location and number of needles were not identified as a prognostic factor [27].

In the current literature there are several series reporting their results on thermal ablation in pulmonary disease of advanced sarcoma, showing promising results in terms of local control and survival rates (Table 1). However, due to the heterogenicity of the disease, most of them is governed by high variability, regarding the size and the number of lesions and the different histologic subtypes of soft tissue and bone sarcoma. More randomized studies and larger series or a registry are needed to establish the role of ablative techniques in sarcoma metastatic disease, especially regarding microwave ablation and cryoablation as well as to demonstrate any possible factors affecting the outcome.

## 3. Thermal Ablation of Liver Metastasis

The application of thermal ablation techniques in both primary (HCC) and secondary liver lesions has been widely investigated in the past, with proven efficacy and efficiency, comparable to surgery [30]. Thermal ablation constitutes an effective alternative treatment for liver metastasis resection, mostly studied in colorectal metastasis, as it is a cost-effective curative option, with tissue sparing and low complication rates [30]. Like in pulmonary metastatic disease, current literature regarding minimally invasive procedures on liver metastases from sarcomas remains limited.

**Figure 2:**
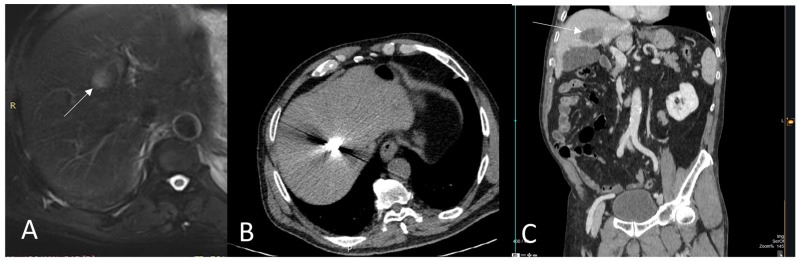
(**A**) A 64 years-old male leiomyosarcoma patient with a solitary hepatic metastasis (white arrow); (**B**) CT-guided microwave ablation was performed; (**C**) Post ablation CT scan, coronal reconstruction (portal venous phase) illustrates the zone of necrosis (white arrow).

Liver constitutes one of the commonest sites of sarcoma metastatic disease, with a prevalence of up to 16% of all patients with retroperitoneal sarcomas and up to 62% of all patients with visceral sarcomas [30]. Systemic therapy remains the standard of care for extrapulmonary metastatic disease whereas surgery and percutaneous ablative techniques are suggested in selected cases (Figure 2), [30]. Liver metastasectomy has been previously described to be a curative treatment option in a limited group of patients [28,31]. Negative prognostic factors constitute the higher tumor grade, the histological type, and the microscopic positive resection margin [31].

Regarding sarcomas, most of the current literature focuses mainly on the treatment of liver metastasis from gastrointestinal stromal tumors (GIST). Gastrointestinal stromal tumor is classified as a soft tissue sarcoma according to the WHO classification; however, since the introduction of the treatment with tyrosine kinase inhibitors, the clinical outcome of gastrointestinal stromal tumors has dramatically improved, prognosis has changed and therefore due to this unique biologic behavior, GIST tumors are usually studied separately [32]. Liver metastases from GIST tumors are relatively often with a wide range between 20–60% [32]. Systemic treatment with chemotherapy is recommended for advanced disease, while metastasectomy should be considered as part of the second- and third-line treatment in selected cases. However, recent studies have highlighted the beneficial effect of surgical resection in the treatment of metastatic GISTs in combination with tyrosine kinase inhibitors administration, reporting 5-year survival rates as high as 91% [33,34]. Moreover, surgery of residual disease upon best clinical response conveys a survival benefit compared with historical controls in patients treated with imatinib alone [35]. Besides surgery, minimally invasive techniques have emerged as an alternative treatment for GIST-related liver metastatic disease. One of the largest retrospective studies available, included 29 patients (66 lesions with a median size of 1.3 cm) who underwent ultrasound guided RFA for liver metastatic disease after failure of the medical treatment with imatinib mesylate [36]. The median overall survival period was 90.2 months (range 12.3–108.6 months) while 4 out of 66 lesions (6%) recurred in an interval period between 3.2 to 10.5 months. Jones et al. [37] performed RFA in 13 GIST patients (12 patients under first-line imatinib therapy and one rechallenged with imatinib) and reported a median time to progression of 26 months. De Baere et al. [25] reported their experience of 17 patients [27 metastasis with a mean diameter of 2.5 cm (range 0.9–4.5 cm)], performing RFA by three different strategies: (A) RFA of all residual tumors during and after imatinib therapy (B) RFA for individual liver metastases progressing under imatinib and (C) RFA of all residual tumours after imatinib therapy, with interruption of systemic treatment. Progression free survival was comparable to surgery in the first two groups, with the advantage of shorter hospital stay and less surgical morbidity.

Excluding GIST, ablation techniques on metastatic liver disease from other sarcoma subtypes, have been mainly studied along with that of other primary tumors. Littrup et al. reported their outcomes of percutaneous cryoablation in 212 patients with 443 liver tumors in total including 49 sarcoma liver metastases of diverse histologic subtypes [38]. While sarcoma-specific data analysis was not performed, the overall local recurrence rate for non-colorectal cancer liver metastases was 9.4% at a mean follow-up of 1.8 years.

Overall, in agreement to surgical studies, literature regarding thermal ablation procedures is characterized by a relatively small number of patients and high heterogenicity regarding the exact histological parameters (Table 2). More randomized trials are needed to investigate the role of ablation in liver metastatic disease, in terms of ablation modality used, possible prognostic factors and the optimal combination with systemic therapy.

## 4. Thermal Ablation of Bone Metastasis

Bone metastatic disease in sarcoma patients approximately affects 2.2% of patients at diagnosis [39]. Although systemic therapy remains the primary treatment, surgery in selected cases can prolong survival [38]. The 5-year overall survival rate of patients with isolated bone metastases ranges between 26.9–54.9% [39]. Historically, ablative techniques have been successfully used in the treatment of benign bone lesions, as well as for curative purposes in oligometastatic patients or as a palliative treatment in symptomatic patients suffering from pain and mobility impairment [40,41]. Deschamps et al. [42] in a large series, including 89 oligometastatic patients with 122 bone metastases, 5 of which were secondary lesions due to sarcoma, performed CT guided RFA or CA and reported a complete destruction rate of 67% at 1 year with 85% complete destruction in metastases with a diameter <2 cm. Prognostic factors decreasing the risk of recurrence and treatment failure were the absence of cortical bone erosion and a maximum diameter lesion <2 cm. Patient characteristics, the site of the primary tumor, previous treatment with radiotherapy, the location of the lesion and the thermal ablation technique used were not associated with the outcome. In the study of McMenomy et al. [43] including 19 oligometastatic patients (4 with sarcoma) with 37 bone metastases, cryoablation was performed, with a complete response rate of 68%. The 1- and 2-years overall survival rates were 91% and 84% respectively.

**Figure 3:**
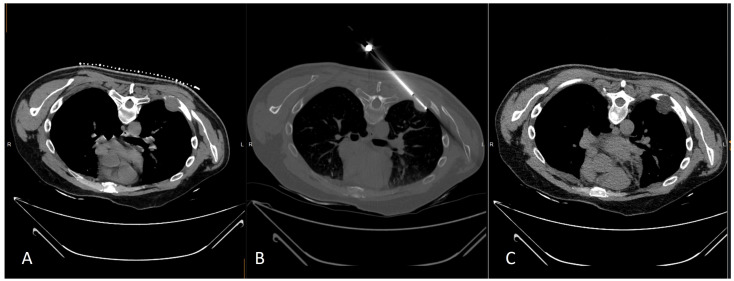
(**A**) CT axial scan illustrating a lytic thoracic wall metastasis in a 46 years-old male hemangiopericytoma patient; (**B**) CT-guided cryoablation was performed for pain palliation; (**C**) Post ablation CT axial scan illustrates the ice ball covering the whole lesion and safety margins.

The role of thermal ablation techniques in palliative care has been widely studied and established as a safe and effective method for primary and secondary bone lesions (Figure 3), [40,41,44].

Vaswani et al. evaluated the impact of percutaneous ablation upon radiographic local tumor control and pain palliation in sarcoma metastases (64 metastatic lesions, 13/64 in oligometastatic patients) within the musculoskeletal system; in this series authors concluded that ablative techniques are an effective option both for local tumor control and pain palliation whilst in the oligometastatic setting can offer potential for remission [45]. Kurup et al. reported outcomes of percutaneous CA in 5 patients with recurrent sacrococcygeal chordomas concluding that it can be considered a safe and efficacious technique for local tumor control and pain palliation in this location and patient population [46].

Additionally, RFA was successfully applied in pediatric population, both for palliative and curative purposes. In the study of Saumet et al. [29] RFA was used in the treatment of osteosarcoma bone metastases for palliation purposes in four cases and with curative intent in three patients with small metastatic lesions in the spine and lower extremities. The authors showed a complete remission at 24 months post-ablation in the total of three patients. Complications were relatively rare in both studies including small hematomas, soft tissue infections and superficial burns. Extra care should be given in spinal lesions to avoid the risk of nerve damage and myelopathy.

As far as soft tissues metastatic disease is concerned outcomes upon all ablative techniques have been reported highlighting that percutaneous ablation is a safe and efficacious therapy for both palliative and curative intent [47,48,49,50]. Once again studies focusing upon soft tissues sarcoma metastatic disease are governed by heterogeneity of the patient populations with respect to histologic subtype, location of treatment, combination to other therapies and ablation modality used. Specifically, though for applied ablation modalities it must be noted that CA is the most reported ablation modality regarding soft tissues sarcoma metastatic disease.

Specifically for osseous and soft tissue metastatic disease MR-guided High Intensity Focus Ultrasound (HIFU) may be an alternative to ablative or surgical techniques achieving local tumor control, pain palliation, and biochemical response particularly in the setting of local tumor recurrence [51,52].

## 5. Conclusions

The metastatic spread of soft tissue and bone sarcoma constitutes a complex and still under investigation scenario, which requires a multi-disciplinary approach. Percutaneous ablation methods may be considered as attractive alternatives or add-on techniques in palliative and curative treatments and should be proposed in tumor board discussions. The role and timing of ablative techniques in the therapeutic toolbox of sarcoma metastatic disease is of outmost importance. Specifically, in the oligometastatic setting ablative techniques can offer potential for remission. Non-thermal techniques such as IRE could overpass limitations in challenging locations, however this needs to be proven and supported by literature data.

The dedicated series in the current literature are limited, mainly retrospective and highly heterogenous rendering comparison of results a difficult task. Therefore, prospective series and randomized trials or a registry focusing exclusively upon sarcoma metastatic disease will further evaluate the role of minimally invasive ablative therapies, their efficacy and prognostic factors affecting the outcome.

## Figures and Tables

**Table 1:** Thermal ablation of lung metastasis from sarcoma.

Author	Type of Ablation	Number of Patients (N)	Numbers of Lesions	Mean Size (mm)	Mean Follow Up (Months)	Mean Overall Survival Rate	Median Progression-Free Survival Rate	Complications
Koelblinger et al. [26]	CT guided RFA	22	55	9	20	94% and 85% in 2 & 3-years	53% and 23% in 1 & 2 years Median time to local tumor progression was 12 months	2 patients with grade III
Nakamura et al. [28]	CT guided RFA	20	89	14 ± 9	18	58% and 29% in 1 & 3 years (medium 12.9 months)	Four of 20 patients (20%) experienced local tumor progression. Median time to local tumor progression was 7.5 months.	38% needed pneumothorax tube
Palussière et al. [19]	CT guided RFA	29	47	9	50	92.2% and 65.2% in 1 & 3 years	Local control rate was obtained in 42 of 47 ablated metastases (89%) Median time to local tumor progression was 7 months	68.7% pneumothorax
Sato et al. [18]	CT guided RFA RFA	46	144	13.5 ± 9.0	16.9	80.6%, 70.1% and 47.1% in 1, 2 & 3-years (medium 31.7 months)	Primary and secondary efficacy rates were 83.5% and 90.0% at 1 year and 76.3% and 81.4% at 2 years	73% grade I 33% grade II
Bourgouin et al. [22]	21 MWA sessions &18 CA	27	65	11	23	100%, 89%, and 82% in 1, 2 & 3-years	For tumors < 1 cm local control rates were 97% and 95% after MWA & 99% and 98% after CA in 1 & 2 years For tumors > 1 cm, 74% and 62% after MWA & 86% and 79% after CA.	44% < Grade III, chest tube placement in 23%
Yevich et al. [21] Pediatric population	CT guided RFA+ CRYO	11	26	6.7	16.7		Five patients remained in complete remission after 37.5 months & five patients developed new metastases	3 pneumothoraxes
Saumet et al. [29] Pediatric population	CT guided RFA	10	22	n/a	24		Median progression-free survival was 21.5 months	3 hemoptysis and 3 pneumothorax

**Table 2:** Thermal ablation of liver metastasis from sarcoma.

Authors	Type of Ablation	Type of Sarcoma	Number of Patients (N)	Mean Lesion Size	Mean Follow Up (Months)	Median Time to Progression	Overall Survival Rate/Time	Complications
Jones et al. [37]	CT guided RFA	GIST	13	-	21	28 months	2-year overall survival was 77%	3 patients with sepsis
Jung et al. [36]	US guided RFA	GIST	29	1.3 cm	33.1	6% showed local recurrence at 3.2 and 10.5 months	90.2 months	1 patient with bleeding at the ablation site & 1 peritoneal seeding near the ablation tract
Littrup et al. [38]	CT guided CA	Various	49	-	20	-	-	

## Data Availability

Not applicable.
